# Greater Omentum Imaging-Reporting and Data System: establishing the grade of benign and malignant lesions of the greater omentum using ultrasonography

**DOI:** 10.1186/s40644-020-00332-z

**Published:** 2020-08-03

**Authors:** Zhiguang Chen, Liang Sang, Yixia Zhang, Donglin Bian, Chunmei Tao, Xuemei Wang

**Affiliations:** Department of Ultrasound, The First Hospital of China Medical University, No. 155, Nanjing North Street, Heping District, Shenyang, 110001 Liaoning Province China

**Keywords:** Omentum, Ultrasound, Greater Omentum imaging-reporting and data system (GOI-RADS)

## Abstract

**Objective:**

To establish Greater Omentum Imaging-Reporting and Data System (GOI-RADS) to evaluate the possibility of omental diseases being malignant.

**Method:**

A retrospective analysis was made of 883 patients who had undergone biopsy of the greater omentum in our center from October 2009 to October 2019. Twelve parameters of ultrasonographic images were evaluated, and the odds ratio of each group calculated. We assigned scores for the direct signs (omental echo, omental structure, and omental nodules) and indirect signs (separation of ascites, echo of ascites, mesenteric lymph nodes, and thickening of parietal peritoneum) of omental lesions. We created an omental score (OS) for each patient and receiver operating characteristic (ROC) curve to analyze its effectiveness in the differential diagnosis of benign and malignant omental diseases.

**Results:**

The OS was divided into ≤5, 6, 7, 8, 9, 10, 11, 12, 13, and ≥ 14 points, and the malignant rate was 0, 1.85, 5.56, 30.36, 37.25, 87.72, 96.72, 98.28, 99.08, and 100%, respectively. The area under the ROC curve (AUC) was 0.976. When taking 10 points as the cutoff value to diagnose benign and malignant omental diseases, the sensitivity and specificity was 93.85 and 98.21%, respectively. A grading system was established: grade 1: omental score ≤ 5, malignant rate 0%; grade 2: omental score 6–7, malignant rate ≤ 5.56%; grade 3: omental score 8-–9, malignant rate ≤ 37.25%; grade 4: omental score ≥ 10, malignant rate ≥ 87.72.

**Conclusion:**

GOI-RADS had high sensitivity and specificity in the differential diagnosis of benign and malignant omental lesions. We believe that GOI-RADS will aid the diagnosis of omental diseases based on objective and accurate interpretation of ultrasound features, and also to promote the ultrasonography of omental diseases in clinical application.

## Introduction

In recent years, there have been improvements in the diagnostic skills of physicians with ultrasound specialization (PWUS) and ultrasonic instrument. Hence, the diagnosis of omental diseases has gradually entered the scope of PWUS. Indeed, the linear array probe has allowed clearer observation of the structure, echo characteristics, blood flow, and small nodules in the omentum. Influenced by the clinical experience of the examiner and resolution of ultrasound instrument, there are considerable differences in the description and diagnosis of omental diseases, which affect disease management. Although elasticity imaging has high sensitivity and specificity in judging benign and malignant lesions of the omentum [1], its basis remains two-dimensional ultrasound.

Nowadays, more and more disease-classification systems have been proposed, such as: Thyroid Imaging-Reporting and Data System [2], Breast Imaging-Reporting and Data System [3], Prostate Imaging - Reporting and Data System [4], and Liver Imaging-Reporting and Data System [5]. Introduction of these concepts has important clinical applications, therefore, as imaging doctors, we should continue to explore new areas of image reporting and data system.

Based on the research and practice of our team over many years, we have found that multiple ultrasound signs of omental diseases may have diagnostic importance. Here, we summarized the relevant signs to judge the nature of omental diseases. Next, we established Greater Omentum Imaging-Reporting and Data System (GOI-RADS) to evaluate the possibility of omental diseases being malignant. In this way, we hope that PWUS can understand benign and malignant diseases of the omentum.

## Materials and methods

### Study cohort

Retrospective analyses were undertaken of the ultrasonograms and ultrasonographic reports of 883 patients who underwent omental examination at the Ultrasonography Department of The First Affiliated Hospital of China Medical University from October 2009 to October 2019. The study cohort comprised 598 women and 285 men (13–88 years; median, 59 years). All 883 patients met the requirements for puncture, and pathology results under ultrasound guidance were obtained. All methods were carried out in accordance with the guidelines set forth in the Declaration of Helsinki. All patients provided oral or written informed consent to participate in our study before their biopsy. Those who are under 18 years, informed consent was obtained from their parent or legal guardian.

### Instruments

The diagnostic instruments we employed were Preirus® (linear array probe = 5–12 MHz; convex array probe = 3–7 MHz; Hitachi, Tokyo, Japan) and AixPlorer® (linear array probe = 4–15 MHz; convex array probe = 3–7 MHz; Supersonic,France).

### Inspection methods

For the ultrasonography of peritoneum, supine position is the main position, scanning up to the xiphoid process, down to the umbilicus. When scanning the peritoneum around the liver or spleen, right or left lateral decubitus position is preferred. The entire abdomen was scanned with the convex array probe. The position of omental lesions, omental thickness, ascites volume, whether there was separation in ascites, and whether the ascites echo was homogeneous were recorded. Then, we switched to the linear array probe, and recorded the echo, structure, and blood flow in the omentum, and whether there were nodules or mesenteric lymph nodes. The depth, gain, and focus of scanning were adjusted according to physical status.

### Criteria for diagnosis (Table 1)

#### Omental thickness

Salman et al. [6] have suggested that omental thickness ≤ 19 mm is a predictor of benign omental lesions. Therefore, we divided omental thickness into > 19 mm and ≤ 19 mm.

**Table 1:** Number of patients with various ultrasound signs and their assignments

Ultrasonic signs	Malignant (Num.)	Benign (Num.)	Odds ratio (OR)	Assignment
	650	233		
Direct sign				
Omental thickness				
>1.90 cm	580	176	2.68	–
<1.90 cm	70	57	0.37	–
Omental display site				
entire	134	65	0.67	–
local	516	168	1.49	–
Omental echo				
hyperechoic	209	98	0.65	1
hypoechoic	347	11	23.11	3
high–low mixed echo	94	124	0.15	0
Omental structure				
moth-eaten	90	3	12.32	3
dense	454	54	7.72	2
loose	105	176	0.06	0
Omental blood flow				
No blood flow	1	1	0.36	–
point-strip	366	178	0.40	–
branch	283	54	2.56	–
Nodules in the omentum				
with	182	14	6.08	2
without	468	219	0.16	0
Indirect sign				
Ascites volume				
none	8	5	0.57	–
small	47	28	0.57	–
medium	384	103	1.82	–
large	211	97	0.67	–
Ascites separation				
with	9	50	0.05	0
without	641	183	19.46	3
Ascites echo				
homogeneous	499	225	0.12	0
heterogeneous	151	8	8.51	2
Aggregation of intestinal tube				
with	26	24	0.35	–
without	624	209	2.89	–
Mesenteric lymph node				
display	25	26	0.3	1
Not display	625	207	3.3	2
Peritoneum wall				
display	64	62	0.3	1
Not display	586	171	3.12	2

#### Omental display site

The omental display site was divided into the “entire display” and “local display” of the abdomen. During scanning, whether there was a thickened omentum in the right-upper, left-upper, right-lower, or left-lower abdomen was documented.

#### Omental echo

**Fig. 1:**
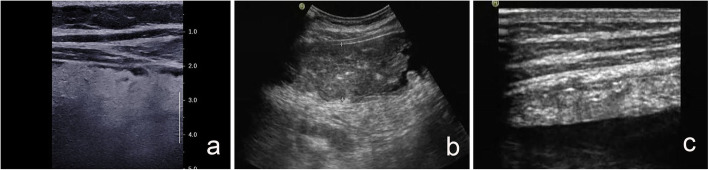
Omental echo. **a** Male, 49 years old, omental echo: hyperechoic. Pathological results: omental tuberculosis. **b** Female, 54 years old, omental echo: hypoechoic. Pathological results: omental metastasis (ovarian cancer metastasis). **c** Male, 23 years old, omental echo: high and low mixed echo. Pathological results: omental tuberculosis

In the disease state, the omental echo can be increased diffusely or be mainly hyperechoic. The latter can include the presence of hypoechoic nodules in a background of hyperechoic signals and the proportion of hyperechoic omentum is > 50% (Fig. 1a), suggesting a hyperechoic scenario. In the disease state, omental echo can be decreased uniformly or be mainly hypoechoic (the proportion of hypoechoic omentum is > 50%), suggesting a hypoechoic state (Fig. 1b). If there is no obvious high or low slice echo area in the thickened omentum, and the proportion of high and low echo of the omentum is close, suggesting a high–low mixed echo (Fig. 1c).

#### Omental structure

**Fig. 2:**
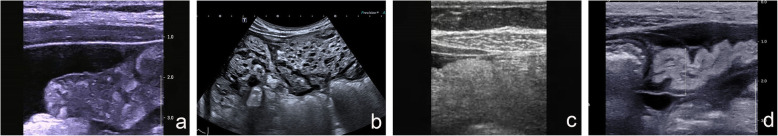
Omental structure. **a** Female, 58 years old, omental structure: moth-eaten. Pathological result: omental metastasis (ovarian cancer metastasis). **b** Female, 63 years old, omental structure: t reticular distribution of a non-echo area in a thickened omentum. Pathological result: omental pseudomyxoma. **c** Female, 71 years old, omental structure: dense. Pathological result: malignant (unclear source). **d** Male, 38 years old, omental structure: loose. Pathological results: omental tuberculosis

A thickened omentum can appear to be “moth-eaten” (Fig. 2a) or there can be a reticular distribution of a non-echo area in a thickened omentum (Fig. 2b). The latter is the characteristic sign of a omental pseudomyxoma. The omental structure can be divided into “dense” (Fig. 2c) and “loose” (Fig. 2d) by imaging using a linear array probe.

#### Omental blood flow

**Fig. 3:**
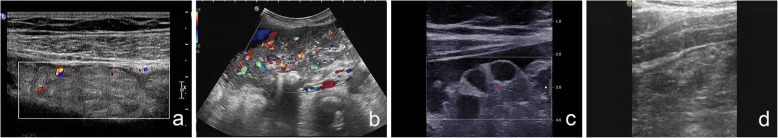
Omental blood flow and nodules. **a** Female, 56 years old, omental blood flow: point-strip blood flow. Pathological results: omental metastasis (ovarian cancer metastasis). **b** Female, 68 years old, omental blood flow: branch blood flow. Pathological results: omental metastasis (ovarian cancer metastasis). **c** Female, 48 years old, omental nodule: multiple. Pathological results: omental metastasis (ovarian cancer metastasis). **d** Male, 65 years old, omental nodule: single pathological result: adenocarcinoma (source of digestive tract)

According to the course of blood flow and different sections of scanning, it can be divided into “point-strip” (Fig. 3a) and “branch” (Fig. 3b) blood flow.

#### Nodules in the omentum

Larger nodules can be found by imaging with a convex array probe. For smaller nodules, a linear array probe is needed, and the nodules can be multiple (Fig. 3c) or single (Fig. 3d). This sign could be divided into “with” or “without” nodules.

#### Ascites volume

A maximum depth of pelvic ascites measured by ultrasound ≤3.0 cm was classified as a “small amount”, 3–10 cm as a “medium amount”, and ≥ 10 cm as a “large amount,” of ascites.

#### Ascites separation

**Fig. 4:**
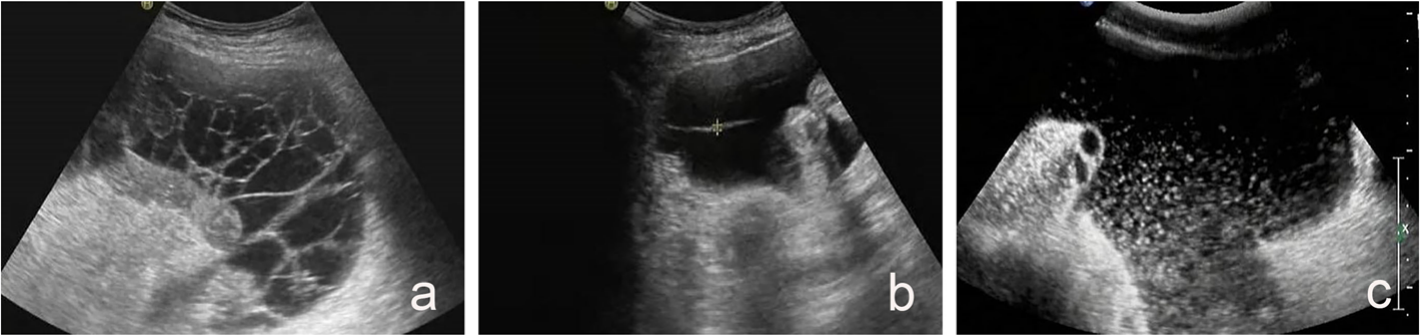
Ascites separation and echo. **a** Male, 39 years old, multiple separation in ascites. Pathological results of omentum: tuberculosis. **b** Female, 25 years old, single separation in ascites. Pathological results of omentum: tuberculosis. **c** Female, 74 years old, with multiple flocculent echoes in ascites. Pathological results of omentum: metastatic carcinoma (ovarian cancer metastasis)

“Strip-like” hyperechoic features in ascites can be observed. It can “float” in ascites and be connected between the inner side wall of the abdomen and mesentery. The separation can be intensive and multiple (Fig. 4a) or single (Fig. 4b). This sign can be divided into “with” or “without” separation.

#### Ascites echo

Ascites echo can be divided into “homogeneous” and “heterogeneous” (Fig. 4c). The former refers to a uniform echoless signal, whereas the latter includes “flocculent echo in ascites” and “gelled ascites.”

#### Aggregation of intestinal tube

**Fig. 5:**
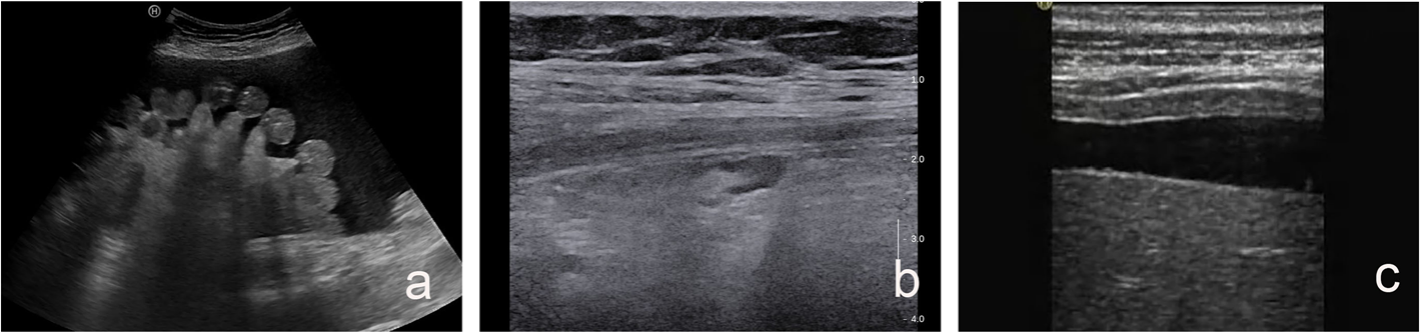
Aggregation of intestinal tube, mesenteric lymph node and peritoneum wall. **a** Female, 66 years old, aggregation of intestinal tube in the left lower abdominal cavity. Pathological results of omentum: metastatic carcinoma (ovarian cancer metastasis). **b** Female, 46 years old, mesenteric lymph node. Pathological results of omentum: inflammation. **c** Female, 62 years old, wall peritoneal thickening. Pathological results of omentum: tuberculosis

Aggregation of intestinal tube refers to aggregation of intestinal tube in a certain area of the abdominal cavity (Fig. 5a), not a single intestinal tract “floating” in ascites.

#### Mesenteric lymph node

Mesenteric lymph node (Fig. 5b) can be divided into “display” and “not display.”

#### Parietal peritoneum

The thickened wall of the peritoneum that can be detected by ultrasound is classified as “peritoneal display” (Fig. 5c). The thinnest peritoneal display in our study was 1.5 mm.

### Assignment method

If odds ratio (OR) was < 0.3, there was no correlation with malignancy. If 0.3 < OR ≤ 3, there was no significant correlation with malignancy. If 3 < OR ≤ 10, there was a strong correlation with malignancy. If OR > 10, there was a extremely strong correlation with malignancy. Of the four scenarios mentioned above, the assignment was 0, 1, 2, or 3 points, respectively.

In the present study, OR of omental thickness (> 19 mm/≤19 mm), omental display site (whole abdomen/local), omental blood flow (dotted strip/branching), ascites volume (none, small, medium or large amount), intestinal-tube aggregation (with/without) were 0.3–3, indicating that there was no significant correlation between those ultrasound signs and nature of omental lesions. The signs of this part are all assigned 1 point, and the results are not statistically significant. So the author excluded and gave up the assignment of this part of signs.

Hence, only the direct signs (omental echo, omental structure, and omental nodules) and indirect signs (whether ascites was accompanied with separation, ascites echo was homogeneous, mesenteric lymph nodes were displayed, and parietal peritoneum was thickened) of omental lesions were evaluated.

### Statistical methods

We used SPSS v22 (IBM, Armonk, NY, USA). Measurement data are the mean ± SD. The number of patients in each group was calculated and assigned after calculation of OR. The assigned value was inputted into the data for each patient, and a final omental score was calculated. The latter was analyzed statistically. A receiver operating characteristic curve (ROC) was drawn to calculate the best diagnostic cutoff point. The proportion of benign and malignant lesions was subdivided. The area under the ROC curve (AUC) was calculated. *P* values were measured using the Student’s *t*-test between each group. *P* < 0.05 was considered significant.

### Importance of the AUC

The AUC, It lies between 0 and 1. The closer the AUC is to 1, the better is the diagnostic effect. The accuracy of AUC is low between 0.5 to 0.7, a certain accuracy at 0.7–0.9, and a higher accuracy at above 0.9. If AUC ≤ 0.5, the method has no diagnostic value.

## Results

### Clinical and pathology results of 883 cases of omental diseases

According to pathology results, omental lesions were divided into 650 cases of malignant lesions group and 233 cases of benign lesions group. The proportion of pathologic types and demographic features are detailed in Table 2.

**Table 2:** Clinical and puncture pathological results of omental lesions in 883 patients

	Number of patients (Num.)	Proportion	Gender		Age		
			Male	Female	Minimum	Maximum	Median
Omental malignancy							
Gynecological disease metastasis	327	50.31%		327	27	85	60
Gastrointestinal metastasis	110	16.92%	77	33	24	86	61
Malignant peritoneal mesothelioma	41	6.31%	31	10	25	81	60
Pseudomyxoma	18	2.77%	13	5	36	79	62
Lymphoma	16	2.46%	12	4	24	76	54
Metastasis of lung cancer	10	1.54%	4	6	45	82	60
Metastasis of breast cancer	7	1.08%		7	44	65	53
Primary cancer	5	0.77%	1	4	57	77	67
Malignant (source unknown)	116	17.85%	26	90	33	82	60
Benign lesions of omentum							
Tuberculosis	184	78.97%	96	88	13	85	51
Inflammation	49	21.03%	25	24	20	88	57

### The diagnostic efficacy of omental score in benign and malignant lesions of omentum

**Fig. 6:**
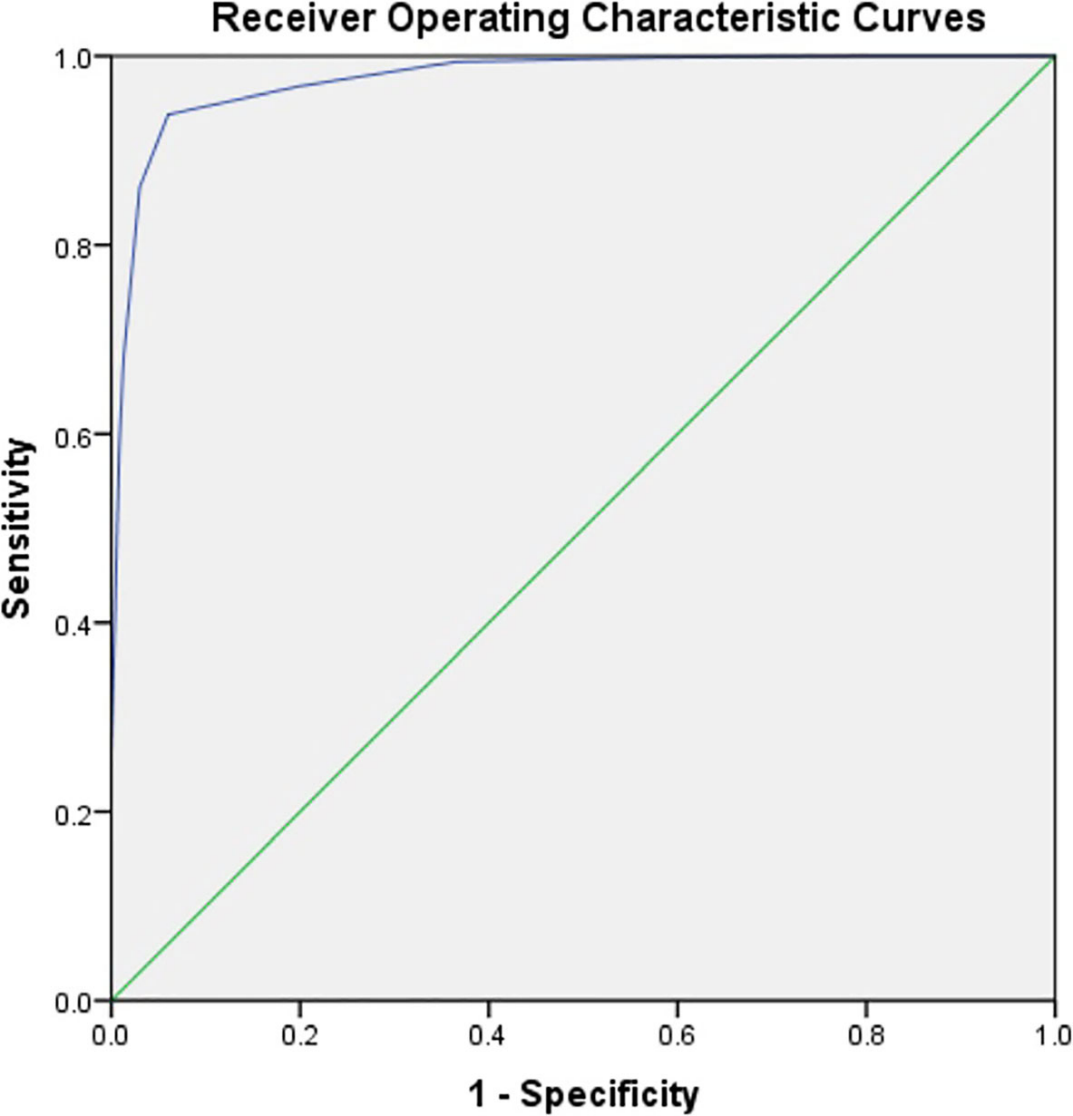
ROC curve of omental score in the diagnosis of omental lesions. The AUC was 0.976

According to the assignment results, we calculated the omental score (OS) of each patient and the malignant rate (Table 3) of different scores. Then, we created a ROC curve (Fig. 6). The best cutoff point for identifying benign and malignant lesions of the omentum was 10 points, with a sensitivity and specificity of 93.85 and 98.21%, respectively.

**Table 3:** Evaluation results and malignant rates of benign and malignant omental lesions

	Omental score (points)									
	≤5	6	7	8	9	10	11	12	13	≥14
Benign	44	53	51	39	32	7	4	1	2	0
Malignant	0	1	3	17	19	50	118	57	215	162
Malignant rate (%)	0	1.85	5.56	30.36	37.25	87.72	96.72	98.28	99.08	100

### Grading system of omental score

According to the results stated above, we preliminarily established a grading system. Grade 1 denoted an OS ≤5, malignant rate of 0%; grade 2: OS of 6–7, malignant rate ≤ 5.56%; grade 3: OS of 8–9, malignant rate of 30.36–37.25%; grade 4: OS ≥10, malignant rate ≥ 87.72%.

### AUC and *P* values for different lesions of omentum

The OS for metastasis of gynecologic disease, gastrointestinal disease, breast disease, lung disease, as well as malignant peritoneal mesothelioma, pseudomyxoma, lymphoma, omental tuberculosis, and omental inflammation were calculated (Table 4). The nine omental lesions stated above were evaluated by inter-group *t*-test, and values for AUC and *P* were calculated (Table 5).

**Table 4:** Proportion of different types of benign and malignant lesions

	Omental score (points)															
	3	4	5	6	7	8	9	10	11	12	13	14	15	16	17	18
Gynecological disease metastasi					2	6	7	20	48	24	117	26	54	14	4	5
Gastrointestinal metastasis				1		5	9	9	31	12	30	4	7	1	1	
Malignant peritoneal mesothelioma						4	1	2	6	6	17	1	3	1		
Pseudomyxoma							1	1	2	4	6	2				
Lymphoma								1	2	3	2	4	1	5		
Metastasis of lung cancer									2		2	2	1			
Metastasis of breast cancer					1				3	1	3		1	1		
Tuberculosis	10	13	14	36	39	36	23	5	4	1	2					
Inflammation	2	1	4	17	11	3	9	2

**Table 5:**
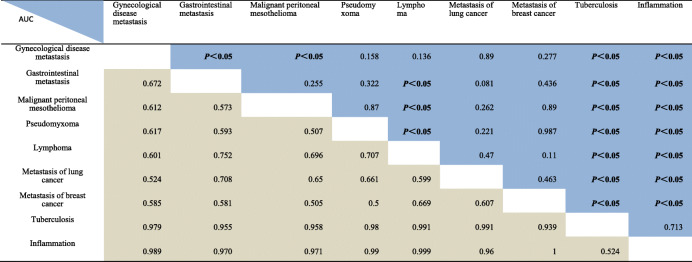
Comparison of omentum scores between nine groups

Explanation: 1 / 2 light blue in the upper right corner of the table is the *p* value of t-test between the two groups; 1 / 2 light gray in the lower left corner of the table refers to theAUC of the two groups. For example: the area under the ROC curve of the first column - the second row was 0.672 represents the AUC of the two groups, at this time, the corresponding *p* value is the first row - the second case < 0.05

## Discussion

The greater omentum is an important part of the peritoneum, and also the largest peritoneal fold in the human body. The greater omentum starts from the great curvature of the stomach and duodenum. The superficial two layers extend downward to the level of the umbilicus and turn back towards the head to form the latter two layers, resulting in four layers of the peritoneum [7].

According to pathology results, omental lesions can be divided into “non-neoplastic” (inflammatory, infectious, reactive), “tumor-like” or “tumor mimics,” and “neoplastic” (primary and secondary) [8]. Most non-neoplastic lesions of the omentum are omental tuberculosis, and the clinical manifestations lack specificity. Most patients with omental disease have ascites, and multiple hyperechoic separation in ascites is a disease feature. The ultrasonogram of a thickened omentum mostly shows two-layer hypoechoic and mixed hyperechoic lesions in the middle. A “simple” hyperechoic lesion is rare, and some patients may have nodules [9, 10]. The most common type of omental neoplasm is due to omental metastasis. The most common source of metastasis is ovarian cancer. The role of ultrasound in tumor staging merits confirmation. Simultaneously, ultrasound has high specificity and sensitivity for the diagnosis of omental metastasis. However, the final diagnosis requires pathologic confirmation [11].

In clinical work, most patients with omental malignant diseases have a clear history of primary tumors, such as lung cancer, breast cancer, ovarian cancer, liver cancer and digestive tract tumor, CT, MRI and PET-CT can directly detect the location of primary lesions and accurately judge the nature of the lesions. In the diagnosis of peritoneal lesions, although most of the imaging manifestations were not specific, those methods had high sensitivity and specificity in the judgment of benign and malignant nature of the lesions [12–14]. However, because CT was not sensitive to lesions with a diameter of less than 1 cm, MRI scan time is long, it is easy to be affected by the motion image, and the cost is high. It is difficult for CT and MRI to distinguish scar tissue after operation and to diagnose omental lesions without obvious masses [15]. For some community hospitals that lack MRI or CT instruments, or for long-term follow-up of patients, ultrasound examination, because of the advantages of no radiation and convenience, fast and affordable, is a desirable auxiliary examination method, which still has certain application value in the monitoring of local lesions and the judgment of the nature of lesions. In addition, for pediatric patients with greater omentum disease, such as greater omentum infarction [16], are more likely to be examined by ultrasonography.

In the present study, direct and indirect signs in the omentum were used as indices to evaluate the nature of omental lesions.

In terms of direct signs, we used:

(i) omental echo (3 points for low echo/1 point for high echo/0 points for high and low echo);
(ii) omental structure (3 points for moth-eaten/2 points for dense/0 points for loose);
(iii) intrareticular nodule (2 points if present/0 point if absent).

With regard to indirect signs, we used:

(i) ascites separation (0 points if present/3 points if absent);
(ii) ascites echo (0 points for homogeneous/2 points for heterogeneous);
(iii) mesenteric lymph nodes (1 point if shown/2 points if not shown);
(iv) parietal peritoneum (1 point for thickening/2 points for not thickening).

By calculating the score of each patient to establish a grading system, the possibility of malignant lesions of the omentum could be predicted objectively by ultrasound signs. The malignant rate increased with increasing OS. If the OS was ≤5, the omental lesion was considered to be benign, with a malignant rate of 0%. If the omentum is inflamed, patients often have abdominal pain, fever, and other symptoms. Therefore, if treatment is timely, the omentum is less affected by inflammatory cells, the echo change of the omental lesion is not obvious, and nodule formation is rare. Moreover, the ascites of patients has reticular hyperechoic separation due to fibrin exudation. Therefore, the OS of patients with omental inflammation or tuberculosis is low [17]. If the OS was ≥14, the malignant rate of omental lesions was 100%. The reason for this finding may be that invasion of tumor cells into the omentum was an aggressive change, and the original hyperechoic structure of the omentum disappeared gradually with disease progression, and it was replaced by a low echoic “nest” of cancer cells, most of which were accompanied by nodules [18]. In addition, ascites was mostly heterogeneous and could be accompanied by bleeding. In patients with pseudomyxoma, the thickened omentum had a reticular distribution of anechoic areas with “jelly-like” ascites, which resulted in a higher OS [19].

For patients with GOI-RADS 1 grade, the malignant rate was 0%. It is not recommended to carry out puncture biopsy. The patients can be regularly followed up by ultrasonography after corresponding clinical treatment (once a month in the first three months, once every three months or half a year after three months). For patients with GOI-RADS 2 grade, the malignant rate was ≤5.56%. CT or MRI can be done first to evaluate whether there is primary focus in other parts of the body. If no primary focus was found, regular ultrasound reexamination can be performed. If abnormal lesions were found, ultrasound-guided greater omentum biopsy can be used to determine the nature of the omental disease. For patients with GOI-RADS 3 grade, the malignant rate was 30.67–37.25%. At this time, ultrasound-guided biopsy of omentum can be performed. For patients with GOI-RADS 4 grade, the malignant rate was ≥87.72%. At this time, if the patient’s physical conditions met the requirements of puncture, it is recommended to carry out ultrasound-guided puncture biopsy. For patients with OS > 14, ultrasound can almost judge the lesions as malignant. It is necessary to carry out ultrasound-guided biopsy at this time. On the one hand, it can confirm the nature of omental lesions and help to locate the primary lesions, so as to find the primary lesions more targeted. Secondly, through the results of immunohistochemistry, the pathological type is clear, which has guiding significance for the selection of clinical treatment plan and prognosis.

Table 5 reveals that the difference between any group of omental malignant disease and any group of omental benign disease is statistically significant, and the AUC is mostly more than 0.9. However, the difference between any inter-group of omental malignant disease and any inter-group of omental benign disease is not statistically significant, and the area under ROC curve is mostly about 0.6.

When liver cirrhosis patients with portal hypertension combined with hepatocellular carcinoma (HCC), peritoneum lesions may be spontaneous bacterial peritonitis (SBP)or the source of HCC metastasis [20–22]. In SBP patients, the omental echo was usually hyperechoic or high-low mixed echo, with loose structure, no nodule, and the ascites echo was mostly homogeneous, which may be accompanied by the display of parietal peritoneum or mesenteric lymph nodes, so the OS was low. While in HCC metastasis to omentum, the omental echo was mostly hypoechoic, dense or wormlike, which may be accompanied by nodule, and the ascites echo was mostly uneven, so the OS is high. Therefore, accurate judgment of benign and malignant omental lesions is conducive to disease management and reasonable allocation of medical resources. We believe that GOI-RADS will aid the diagnosis of omental diseases based on objective and accurate interpretation of ultrasound features. The main limitation of ultrasonography using GOI-RADS was imaging of the omentum in obese people due to obscuring of omental lesions because of excessive adipose tissue.

## Conclusions

Ultrasonography can be employed to reveal an abnormally thickened omentum. We established a grading system of high sensitivity and specificity for differentiation of benign and malignant lesions of the greater omentum: GOI-RADS.

## Data Availability

The datasets generated during and/or analyzed during the current study are available from the corresponding author on reasonable request.

## Abbreviations

AUC: Area Under the receiver operating characteristic Curve
GOI-RADS: Greater Omentum Imaging-Reporting and Data System
HCC: Hepatocellular carcinoma
SBP: Spontaneous bacterial peritonitis
OR: Odds ratio
OS: Omental Score
ROC: Receiver Operating Characteristic
PWUS: Physicians With Ultrasound Specialization
